# Health benefits, antimicrobial activities, and potential applications of probiotics: A review

**DOI:** 10.1097/MD.0000000000032412

**Published:** 2024-12-27

**Authors:** Amin Sepehr, Seyedeh Tina Miri, Shadi Aghamohammad, Nazanin Rahimirad, Mahnaz Milani, Mohammad-Reza Pourshafie, Mahdi Rohani

**Affiliations:** a Department of Bacteriology, Pasteur Institute of Iran, Tehran, Iran; b Department of Microbiology, Islamic Azad University Science and Research Branch, Tehran, Iran; c Department of Genetics, Faculty of Biological Sciences, Tarbiat Modares University, Tehran, Iran.

**Keywords:** antimicrobial activities, functional foods, gastrointestinal cancer, gut microbiota, probiotics

## Abstract

Gut microbiota and its metabolic activities can influence the physiology and pathology of the human body. It is well established that alterations in the balance of living microbiota can contribute to various health problems, such as inflammatory bowel disease and autoimmune disorders. Probiotics administered in sufficient quantities as functional food ingredients provide health benefits to hosts. They help to maintain the stability and composition of the gut microbiota and provide resistance to infection by pathogens. The most important probiotic bacteria are *Lactobacillus* spp. and *Bifidobacteria* spp., which protect the intestine through various mechanisms such as the production of organic acids and bacteriocins. Scientific and clinical research has demonstrated that probiotics play a role in modulating immune response and preventing cancer and chronic inflammatory diseases, especially in the gastrointestinal tract. This article summarizes the potential health benefits, antimicrobial activities, and purposes for which probiotics can be used as functional foods to improve human health.

## 1. Introduction

### 1.1. Probiotics and functional foods

The intestinal microbiota consists of a bacterial community whose metabolic activity can influence the host physiology and pathology.^[1]^ Probiotics are part of the normal gut microbiota and have several beneficial functions, including nutrition (fermentation of indigestible substrates, production of short-chain fatty acids, absorption of ions, and production of amino acids and vitamins), protection (prevention of microbial invasion), immunity (development and homeostasis of local and systemic immunity), and reduction of inflammation by influencing different signaling pathways.^[2,3]^

The word probiotic (literally, “for life”) was introduced in 1953 by German scientist Werner Kollath to refer to “active substances that are essential for the healthy development of life.” In 1965, the term was used in a different context by Lilly and Stillwell to refer to “substances secreted by one organism that stimulate the growth of another.” Fuller defined probiotics in 1992 as “a live microbial food supplement that has a beneficial effect on the host animal by improving its gut microbial balance.”^[4]^ Finally, the Food and Agriculture Organization of the United Nations and the World Health Organization define probiotics as “live microorganisms that, when administered in sufficient quantity, provide health benefits to the host.”^[5,6]^ However, it should be noted that not all probiotics are useful under all circumstances. The careful selection of specific organisms based on the desired clinical outcome is an effective strategy for selecting the appropriate therapy for a disease.^[1]^ Probiotic organisms require specific characteristics to enable them to exert maximum beneficial effects, including gastric acid and bile salt stability, ability to adhere to the intestinal mucosa, colonization of the intestinal tract, and antimicrobial effects against pathogens.^[7,8]^

On the other hand, functional foods, also called nutraceuticals, are similar to ordinary foods we eat every day. However, in addition to their nutritional value, functional foods also provide health benefits that lead to a lower risk of chronic diseases. Probiotics and their derivatives play an essential role in the development and expansion of functional foods and the food industry.^[9,10]^ Nowadays, consumers are aware of the relationship between lifestyle, diet, and health, which explains the increasing demand for products that can improve health by providing essential nutrients.^[11]^ This review aims to present the health benefits, antimicrobial activities, and potential applications of probiotics. In addition, the purpose of this review is to explain some characteristics of 2 important representatives of probiotics (*Lactobacillus* and *Bifidobacterium*) and to discuss the effectiveness of probiotics against pathogens, gastrointestinal (GI) cancer, and inflammation.

### 1.2. Characteristics and application of probiotics

Resistance to gastric and bile acids are essential properties of probiotic strains. It has been suggested that a desirable probiotic must have the ability to withstand a pH of at least 3.0, as this is a common standard to study the acid tolerance of a probiotic culture.^[12]^ Other properties of probiotics include adherence to mucus and/or human epithelial cells and cell lines, antimicrobial activity against potentially pathogenic bacteria, ability to reduce adhesion of pathogens to surfaces, bile salt hydrolase activity, and enhanced viability of probiotics.^[13]^

As mentioned earlier, probiotics have many health benefits, such as an antimutagenic effect, stimulation of the immune system, lowering of blood ammonia levels, lowering of serum cholesterol, strengthening of mucosal barriers, and prevention of intestinal inflammation. In addition, these bacteria reduce lactose intolerance, relieve the symptoms of diarrhea, promote the synthesis of B vitamins, and have antimicrobial activity against various pathogens, such as *Helicobacter pylori*, *Escherichia coli*, *Clostridium* spp., *Salmonella* spp., and *Campylobacter* spp.^[14–16]^

It has been demonstrated that the antimicrobial activity of probiotics can be achieved through different strategies. For example, probiotics can protect against pathogens by producing bacteriocins, lactic acids, and chemical compounds such as H_2_O_2_ and also prevent bacterial colonization.^[17]^ Under normal conditions, careful regulation limits excessive inflammation and maintains immune system balance, which in turn increases susceptibility to chronic inflammatory disease. The disruption of the interactions between diet, microbiome, and host organism is commonly referred to as “dysbiosis” and is a recurring element of several chronic inflammatory diseases.^[18]^ Multiple strains of probiotics have been shown to exert diverse effects on the host and immune system. Their essential role in regulating inflammation has been well elucidated in several in vitro and *ex vivo* models.^[18,19]^ Moreover, studies have reported that probiotics can bind to a variety of mutagens, such as Trp-*P*-1 (3-amino-1,4-dimethyl-[5H] pyrido [4,3-b] indole) and Trp-*P*-2 (3-amino-1-methyl-[5H]-pyrido[4,3-b]indole),^[19,20]^ making them ineffective. In addition, probiotics have been suggested to be helpful in dietary detoxification.^[21]^ Peltonen et al investigated the ability of probiotics to bind a common food carcinogen, aflatoxin B-1. They suggested that the application of this phenomenon in the removal of mycotoxins from contaminated food is urgently needed to improve the safety of food supply.^[22]^

The probiotics used in this study are summarized in Table 1. In this context, *Lactobacillus* and *Bifidobacterium* are 2 important known probiotics, which are described in more detail below.

**Table 1:** Common microorganisms considered as probiotics.

Family/genus	Species
*Lactobacillus*	*L casei Shirota*, *L rhamnosus GG*, *L johnsonii*, *L acidophilus*, *L gasseri*, *L reuteri*, *L casei*, *L fermentum*, *L crispatus*, *L plantarum*, *L paracasei*, *L bulgaricus*
*Bifidobacteriacea*	*B longum*, *B bifidum*, *B infantis*, *B lactis*, *B breve*, *B animalis*, *B adolescentis*, *B catenulatum*
Other	*Escherichia coli Nissle*, *Saccharomyces boulardii*, *Enterococcus faecalis*, *Lactococcus lactis*, *Propionibacteria*

### 1.3. Characteristics of *Lactobacillus*

*Lactobacillus* is a gram-positive, nonspore-forming, catalase-negative, and lactic acid-producing bacterium with limited biosynthetic ability that can ferment lactic acid as an end product.^[23]^ *Lactobacillus* is the largest of the 3 genera within the family *Lactobacillaceae*, and belongs to one of the dominant phyla, Firmicutes. Furthermore, *Lactobacillus* is typically divided into 3 groups based on various fermentative pathways and 7 groups based on their *16S rRNA* gene sequences.^[24]^ *Lactobacillus* species are essential members of the human and other mammalian normal microbiota and are defined as the best species used as probiotics.^[23]^ Historical evidence of the beneficial effects of *Lactobacillus* as a probiotic strain came from Rettger and Cheplin in 1920, who experimented with *Lactobacillus acidophilus* to study its effects on the growth of pathogenic bacteria.^[25]^ Studies on human and mouse models have demonstrated that immune regulatory and potential probiotic roles limit diverse infectious, allergic, and inflammatory diseases. The probiotic characteristics of many *Lactobacillus* strains confirm their competitive role against pathogens in attaching epithelial cells, producing antimicrobial peptides, and helping epithelial integrity.^[26]^ Many studies on different isolates of *Lactobacillus* supported this efficacy. For example, *Lactobacillus casei* has been used as a probiotic to reduce the incidence of diarrhea in children and alleviate inflammatory symptoms. In addition, *Lactobacillus plantarum* has been shown to suppress inflammation in patients with inflammatory bowel disease (IBD) and regulate gut microbiota. These strains suppress the growth of *Shigella*, *Salmonella* spp., *E coli* and other intestinal pathogens, and cure intestinal diseases with fewer side effects than antibiotic therapy.^[27,28]^

### 1.4. Characteristics of *Bifidobacterium*

*Bifidobacterium* is a gram-positive, heterofermentative, nonmotile, and non-spore-forming bacterium belonging to the phylum *Actinobacteria*. The most recent *Taxonomic Outline of Bacteria and Archaea*, release 7.7, suggests that the *Bifidobacteriaceae* family should be divided into 5 genera: *Bifidobacterium*, *Aeriscardovia*, *Gardnerella*, *Parascardovia*, and *Scardovia*.^[28]^ The genus *Bifidobacterium* belongs to the *Actinomycetaceae* family,^[29]^ with >30 species identified so far, including the most important of them namely *Bifidobacterium lactis* and *Bifidobacterium longum*.^[30]^

In 1899, Henri Tissier first isolated *Bifidobacterium* from the feces of breast-fed infants.^[31]^ This bacterium has originated from a wide variety of ecological environments, of which 3 are directly linked to the human and animal intestinal environment: for example, such as the human gut, animal intestine (bovine, rabbit, murine, chicken, and insect), and oral cavity. However, other species isolated from sewage (*e.g*., *Bifidobacterium minimum* and *Bifidobacterium thermacidophilum*), human blood (*Bifidobacterium scardovii*), and food products (*e.g*., *Bifidobacterium. animalis subsp. lactis*) is likely a consequence of contamination from the GI tract.^[31]^ In 2017, Eshaghi et al assessed native *Bifidobacterium* isolated from human breast milk and feces of paired infants and reported universal anti-pathogenic activity in all isolates.^[32]^ Members of this genus have a long history of use as health-promoting/probiotic strains because of traits such as regulation of intestinal microbial homeostasis, inhibition of pathogen growth, modulation of local and systemic immune responses, maintenance of GI barrier function, production of vitamins, and bioconversion of some dietary compounds into bioactive molecules.^[33]^

## 2. Antimicrobial activities of probiotics

### 2.1. Production of organic acids

Another concept of antagonistic activity of lactic acid bacteria against pathogenic strains is mainly attributed to the production of antimicrobial substances or metabolites, such as organic acids. Organic acids are short-chain fatty acids that play an important role in the efficacy of probiotic isolates. With the help of these substances, probiotics compete with pathogens for nutrients and attachment sites, preventing the colonization of the intestine by pathogens.^[34]^

It has been reported that organic acids, mainly lactic and acetic acids are the most common antimicrobial substances among the antimicrobial agents.^[35]^ The organic acid profiles of *Lactobacillus* strains showed that lactic acid was the predominant acid produced by all strains, followed by acetic acid and succinic acid in lesser amounts. Other acids, namely propionic acid, butyric acid, isobutyric acid, valeric acid, and isovaleric acid, were either not produced or were produced only in trace amounts by some of the strains. Studies have shown that lactic and acetic acids are the major organic acids involved in the antimicrobial activity of *Lactobacillus* strains.^[36]^ Other *Lactobacillus* strains isolated from different origins with potential probiotic and antimicrobial activities against pathogens include *L acidophilus HM1*, *Lactobacillus fermentum HM2*, *L fermentum HM3* (human milk), *L casei BF1*, *L casei BF2*, *L casei BF3* (infant feces), *Lactobacillus buchneri FG1* (fermented grapes), *L buchneri FD1*, and *L buchneri FD2* (fermented dates).^[30]^

Studies have shown that many *Lactobacillus*-based probiotics have been selected based on organic acid production.^[36]^ Sugar fermentation leads to a decrease in pH via the production of organic acids. This process is critical for the inhibition of the growth of undesirable microorganisms. Indeed, an acidic environment makes organic acids lipid-soluble and causes them to cross the pathogen’s cell membrane and reach the cytoplasm.^[37]^ Lactic acid is produced in 2 ways: by chemical synthesis or microbial fermentation. Optically pure L (+) or D (−) lactic acid isomers can be obtained by microbial fermentation using *Lactobacillus* strains. The strains that produced the L (+) lactic acid isomer were *Lactobacillus delbrueckii*, *Lactobacillus amylophilus*, *Lactobacillus bavaricus*, *L casei*, *Lactobacillus maltaromicus*, and *Lactobacillus salivarius*. Strains such as *Lactobacillus lactis*, *Lactobacillus jensenii*, and *L acidophilus* produce either the D (−) isomer or a mixture of both.^[37]^ The *ldhD* gene produces D-lactate dehydrogenase, which is responsible for releasing lactic acid by the catalytic activity ((*R*)-lactate^+^NAD^+^ = H^+^+ NADH^+^ pyruvate) of *Lactobacillus* strains (*L plantarum* [*strain ATCC BAA-793/NCIMB 8826/WCFS1*]).^[38]^ Acetic acid is another major organic acid produced by *Lactobacillus* strains. Several studies have revealed that strains such as *L buchneri* and *Lactobacillus parabuchneri* can convert lactic acid to acetic acid under anoxic conditions. Moreover, *L plantarum* and *Lactobacillus pentosus* can degrade lactic acid under anoxic conditions in the presence of citrate as an electron acceptor, to produce succinic acid, acetate, formate, and CO_2_. In contrast, *Lactobacillus brevis and L buchneri* degrade lactic acid using glycerol as an electron acceptor to produce acetate, 1,3-propanediol, and CO_2_.^[39]^

These findings revealed that *Lactobacillus* could dominate the metabolic activity of *C jejuni* through the production of inhibitory organic acids when the 2 organisms were grown in the same environment.^[40]^ *L delbrueckii* UFV H2b20 is another probiotic strain in which organic acid production during growth in skimmed milk was analyzed. The production of organic acids in industrial food fermentation processes leads to food preservation, flavor formation, and lipid and protein catabolism.^[41]^

*Bifidobacterium* strains may provide various health benefits such as antimicrobial effects. Some strains inhibit the growth or cell adhesion of pathogenic bacteria, including multidrug-resistant bacteria, and their antibacterial activity can be enhanced when combined with certain antibiotics.^[42]^ Makras and Vuyst found robust antibacterial activity of *Bifidobacteria* against gram-negative bacteria, including *Salmonella* and *E coli* strains.^[43]^ In another study, the use of *Bifidobacterium* in the agar plate spot test showed high antimicrobial activity against various pathogenic bacteria due to the production of organic acids.^[44]^ Some *Bifidobacterium* strains, such as *Bifidobacterium adolescentis*, *Bifidobacterium pseudocatenulatum*, and *B longum*, have been found to inhibit the growth of pathogens, including vancomycin-resistant *S aureus* and vancomycin-resistant *Enterococcus*.^[45]^ Another study by Yang et al showed that the antibacterial activity of *Bifidobacterium breve* against Clostridium *difficile* was enhanced when combined with antibiotics such as metronidazole, clindamycin, and ceftazidime.^[46]^

Overall, these results indicate the efficacy of *Lactobacillus* and *Bifidobacterium* strains as potential therapies for infectious diseases. For *Lactobacillus* and *Bifidobacterium* strains to be used for pharmaceutical purposes, further in vivo studies should be conducted to ensure the efficacy and safety of these products in the human body.

### 2.2. Production of bacteriocins

Bacteriocins are antimicrobial peptides produced by bacteria that are active against other bacteria, either within the same species (narrow spectrum) or across genera (broad spectrum).^[47]^ Bacteriocins are currently classified according to molecular mass, thermal stability, and amino acid composition (Table 2).^[49,50]^ Lacticin, lactocin, pisciolin, plantaricin, and nisin are the most common bacteriocins. They are usually sensitive to proteases owing to their protein structure. These compounds perforate the bacterial cell membranes, leading to cell death.^[51,52]^

**Table 2:** Classification of bacteriocins.^[48]^

Bacteriocins classification	Sub class	Definition based on molecular mass and peptide form
Class I	Class Ia	Lanthipeptides
	Class Ib	Head-to-tail cyclized peptides
	Class Ic	Sactibiotics
	Class Id	Linear azol(in)e-containing peptides
	Class Ie	Glycocins
	Class If	Lasso peptides
Class II	Class IIa	Pediocin-like bacteriocins
	Class IIb	Two-peptide bacteriocins
	Class IIc	Leaderless/cyclic bacteriocins
	Class IId	Non-pediocin-like, one/single-peptide bacteriocins
Class III		Unmodified bacteriocins

Studies have shown that a positive effect of *Bifidobacteria* on the human GI tract is the production of antimicrobial compounds other than acids, such as bacteriocins^[53]^ and bacteriocin-like inhibitory substances. Bacteriocinogenic strains of *Bifidobacterium* spp. are of great importance for food safety applications, especially in the dairy industry. *Bifidobacteria* usually exhibit their highest antimicrobial activity at the end of the logarithmic growth phase and/or beginning of the quiescent phase. This phenomenon often occurs due to quorum-sensing factors or environmental stresses imposed on the bacteria and is known as probiotic characteristics.^[54]^ It has been demonstrated that *Bifidobacterium bifidum* NCFB 1454 produces bacteriocin B, which can bind to the cell wall receptor of gram-positive bacteria. Therefore, it destroys these molecules via a pH-dependent mechanism.^[55]^ In addition, bifidiocin B isolated from the *B. animalis* BB04 strain exhibited similar antimicrobial activity by causing lysis of the bacterial cell walls. Acidocin A is another bacteriocin produced by *Bifidobacteria*, that can inhibit *Clostridium* species found in fermented food products.^[56]^ It has also been shown that antibiotic-sensitive or antibiotic-resistant *H pylori* strains are sensitive to heat-resistant bacteriocins isolated from *Bifidobacteria*.^[48]^

Moreover, bacteriocins show antagonistic activity against various pathogens, such as *Listeria monocytogenes*.^[57]^ They also function as signal peptides that communicate with other bacteria via quorum sensing and crosstalk mechanisms between the bacterial population and host immune system cells. Additionally, *B thermophilum* RBL67 can produce a bacteriocin-like inhibitory substance or “lantibiotic.” Whole-genome analyses of *B longum* subsp. *longum* revealed a complete lantibiotic gene cluster.^[58]^

The first *Bifidobacterium*-associated bacteriocin was bifidin produced by *B bifidum NCDC* 1452.^[59]^ Later, Kang et al identified a *B longum* strain^[60]^ with an unknown compound with antimicrobial activity against several gram-positive and gram-negative bacteria called bifilong. Meghrous et al found over 70 compounds with antibacterial properties in *B bifidum* culture medium.^[61]^ In 2000, Liévin et al isolated a lipophilic low-molecular-weight compound with antimicrobial activity against *S typhimurium*.^[62]^ In 2003, Touré et al isolated a *Bifidobacterium* strain with inhibitory effects on *L monocytogenes* from infants.^[63]^ Other studies have reported the isolation of Bifilact Bb-12 and Bifilong Bb-46 from *B lactis*.^[64]^ Antimicrobials from 6 *Bifidobacterium* strains exhibit broad inhibitory spectra against gram-negative and gram-positive bacteria, namely *C difficile*, *B thermosphacta*, *L monocytogenes*, *S aureus*, *H pylori, S. typhimurium, Arcobacter butzleri*, as well as some pathogenic yeasts.^[65,66]^

The amino acid sequences of some bacteriocins isolated from *Bifidobacteria*, such as bifidocin B, bifidin I, and lantibiotic bisin, showed unique properties such as stability under acidic conditions and resistance to weak organic solvents, freezing, and enzymes.^[67]^ Bifidin I was first purified from *Bifidobacterium infantis* BCRC 146021 by Cheikhyoussef in 2010. Evaluation of the antimicrobial activity of bifidin I showed that it had an inhibitory effect of 95% against a wide range of bacteria.^[68]^ In addition, other studies have shown the antimicrobial effect of bisin against some indicator strains of the genera *Bacillus* spp., *Serratia*, and *Streptococcus*.^[69]^

Several mechanisms have been suggested for the inhibitory action of *Bifidobacteria* on gram-negative pathogens. These mechanisms include a decrease in local pH by the production of organic acids, inhibitory activity of undissociated organic acid molecules, competition for nutrients, competition for adhesion sites, stimulation of host immunity, and production of specific antibacterial substances.^[70–72]^

The *B longum* NCC2705 strain can be considered the first sequenced species of commensal bacteria. After publishing the sequence of this bacterium in the corresponding database, a complete sequence of the strain *B. longum* DLO10A (GenBank accession number: NZ_AABM00000000) and other studied strains were presented to the public.^[73]^

Lantibiotics are a broad spectrum class of bacteriocins. They differ from other bacteriocins in that they contain posttranslationally transformed amino acids, such as lanthionine and methyllanthionine, and cyclic structures.^[74]^ The first bifidobacterial lantibiotic purified from *B longum* subsp. *longum* DJO10A was encoded by a characteristic lantibiotic operon with genes encoding production, modification, and regulatory functions.^[69]^ This operon consists of genes encoding a two-component signal transduction system (*lanR2* and *lanK*), a lantibiotic pre-peptide (*lanA*), a lantibiotic response regulator (*lanR1*), lantibiotic modification enzymes (*lanD* and *lanM*), a lantibiotic immunity protein (*lanI*), and a lantibiotic transporter with predicted protease activity for the removal of the leader peptide during secretion (*lanT*). The comparative genome analysis of *Bifidobacteria* also showed the homologous remnants of this lantibiotic operon in the genomes of *B longum* subsp. *infantis* ATCC 15697 and *B angulatum* DSM 20098, suggesting the possibility of widespread lantibiotic production in *Bifidobacteria*.^[69]^

As reported by Bibalan et al, there are a variety of bacteriocin genes in *Lactobacillus* species. They assessed bacteriocin genes in *Lactobacillu*s strains isolated from healthy individuals and reported that approximately 40% of all *Lactobacillus* isolates were positive in bacteriocin phenotypic tests (antibacterial activity).^[75]^ Hegarty et al isolated 2 new bacteriocin clusters belonging to the antibiotic class from 2 *Bifidobacterium* species.^[76]^ The 6-gene cluster, identified based on the presence of the Lan L-type lanthionine gene, can predict the production of a type of lanthipeptide from the Lan L class. This cluster includes several genes related to lantibiotics, including genes encoding lanthionine, oligopeptidase, and ABC transporter type. In addition, this cluster consists of a 2-component regulatory system comprising a histidine kinase (conserved domain CGO4585 6.70e-18) and an immune system regulator (conserved domain COG2197 8.85e-57).

**Figure 1.**
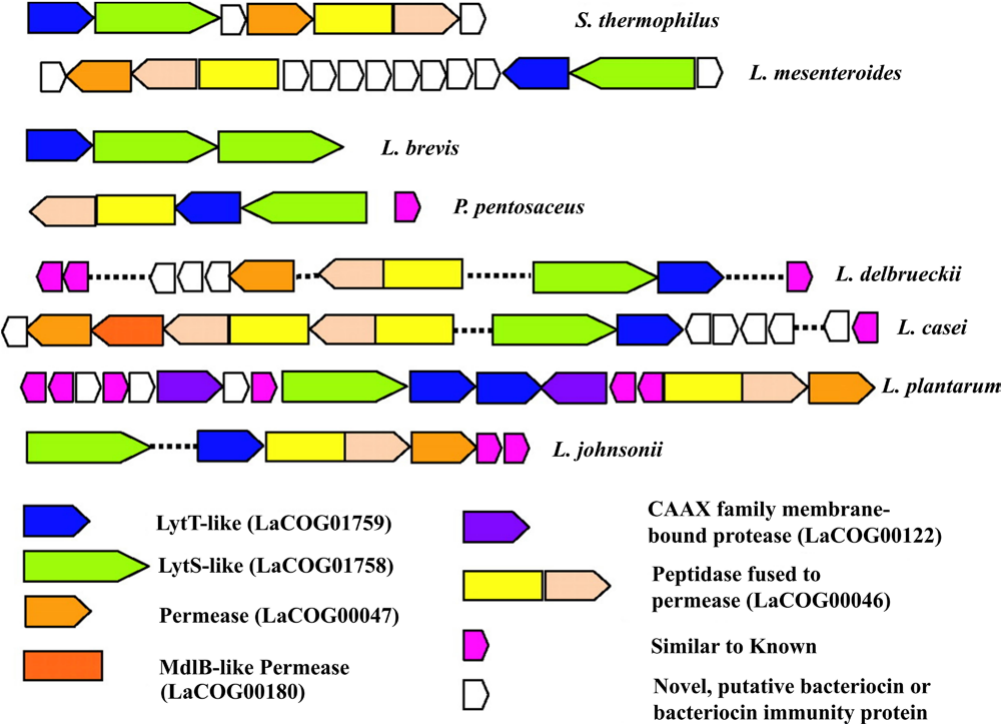
Different gene clusters of *Lactobacillales* encoding bacteriocins. Retrieved from reference^[77]^ with permission of the author.

A lantibiotic with a length of 7966 bp consisting of 6 genes has been identified in the genome of *Bifidobacterium* sp. 12_1_47BFA, with properties similar to those of the lantibiotic Lan A. This gene cluster encodes different compounds, such as Lan M (conserved domain cd04792 0.0) and an ABC-type bacteriocin/lantibiotic exporter (conserved domain COG2274 7.59e-145). This gene cluster also encodes FMN-dependent reductase (conserved domain pfam03358 5.13e-09), and this class of proteins is generally known as unusual post-transcription-modified lantibiotics (Fig. 1).^[77–79]^

## 3. Preventive role of probiotics in GI cancer

Cancer is a serious health concern and is one of the most significant causes of morbidity and mortality worldwide. Carcinogenesis is a multifactorial mechanism with diverse interactions between genetic and epigenetic factors, as well as multiple environmental factors such as diet, nutrition, and lifestyle. Probiotics affect the immunological condition of the organism and function during tumorigenesis and growth^[80,81]^ by affecting susceptibility to immune-mediated and infectious diseases.^[82–84]^ Growing body of research has investigated the correlation between the intestinal microbiota and tumorigenesis, neuropathic conditions, obesity, diabetes, and GI disorders. The effect of these probiotics has been revealed on prevention, treatment, and reducing the progression of cancers.^[85,86]^ Among various cancers, GI cancer refers to malignancies of the GI tract and accessory digestive organs, including the esophagus, stomach, biliary system, pancreas, small intestine, colon, rectum, and anus,^[87,88]^ which account for 25% of all cancers and 9% of all causes of cancer-related death worldwide.^[89]^

Studies have reported the antiproliferative or pro-apoptotic role of probiotics against GI cancers, among which colon and gastric cancers are the most studied.^[90]^ It has been revealed that *L. casei* and *L. acidophilus* increase apoptosis in tumor cells.^[91,92]^ In addition, the *Lactobacillus. rhamnosus* GG strain has an antiproliferative role in gastric and colon cancers.^[93]^ Based on an in vitro study, a probiotic product named *B adolescentis* SPM0212 showed high antiproliferative activity and inhibited human gastric cancer cells in 3 human colon cancer cell lines.^[94]^ Studies have also demonstrated that other probiotic products or strains, such as *Bacillus polyfermenticus* (18), *L acidophilus* 606,^[95]^ *L rhamnosus* GG (LGG)/Bb12,^[96]^ and LGG/*Bifidobacterium animalis* subsp. *lactis*^[97]^ have antitumor activities against human colon cancer. Moreover, another study reported that *Propionibacterium freudenreichii* improved the cytotoxicity of camptothecin as a chemotherapeutic agent for gastric cancer.^[98]^

Probiotics can inactivate carcinogens and alter cellular differentiation via immunomodulatory effects.^[19]^ They stimulate immunogenic elements and improve the activity of the gut barrier by secreting anticarcinogenic and anti-oxidative molecules, which in turn activate mononuclear cells and lymphocytes and upregulate immunoglobulin A.^[19]^ Probiotic administration also reduces the expression of specific toll-like receptors (TLRs), which increase epithelial barrier resistance.^[99]^ This phenomenon activates phagocytes, which contribute to maintaining vigilance against tumor cells, especially in the early stages of progression.^[100]^

## 4. Preventive role of probiotics in colorectal cancer

Colorectal cancer is the third most common malignancy in Asia and developing regions.^[101]^ Studies have reported increased mortality rates in the last decade in Asian countries, including Korea, China, and Iran. Colorectal cancer (CRC) is considered a Westernized diet disease, in which a high-fat and low-fiber diet (the so-called Western diet) is common.^[101–103]^ In recent years, there has been increasing interest in the effect of probiotics in the prevention and inhibition of colorectal carcinogenesis. Patients with colorectal carcinoma have been found to have a decrease in fecal *B animalis* and *S thermophilus*, which produce lactic acid.^[104]^

To maintain homeostasis, the GI tract must adapt to drastic changes in the luminal environment, and responses to ingested food are the most basic physiological adaptations. The epithelial cells (EC) of the GI tract renew rapidly, with a new intestinal lining formed approximately every 3 days. However, turnover rate is not always constant. For example, the proliferation rate of intestinal EC in rodents and humans is subject to circadian variations, which alter the morphology and function of the GI tract.^[105]^ Physiologically generated reactive oxygen species (ROS) are known to function as secondary messengers that influence cellular proliferation and differentiation in various biological systems. Jones *et al* reported that commensal bacteria, particularly members of the genus *Lactobacillus*, can stimulate NADPH oxidase 1 (Nox1)-dependent ROS generation and, thus, cellular proliferation in intestinal stem cells after initial uptake into the mouse or Drosophila intestine.^[106]^

Nonsteroidal anti-inflammatory drugs (NSAIDs) can cause serious GI side effects. Intestinal microorganisms are necessary for the development of NSAID-induced small-bowel lesions. Therefore, it has been suggested that probiotics may protect against NSAID enteropathies. A clinical trial showed that a probiotic mixture significantly lowered fecal calprotectin concentration in healthy volunteers compared to placebo, suggesting that this approach may be useful in reducing indomethacin-induced intestinal inflammation.^[107]^

Recent research has reported significant antiproliferative and apoptotic effects of *Lactobacilli* cocktail on the human colorectal carcinoma cell line HT-29 by affecting genes associated with the Notch and Wnt/β-catenin pathways.^[108]^ Studies have suggested considerable “protective” anti-cancer and significant anti-CRC effects of *Bifidobacterium* species in CRC mice models.^[109]^ In general, this potential probiotic could be considered a suitable dietary supplement for the treatment and prevention of CRC. In addition, secretory molecules of the probiotic cell-free supernatant of *Lactobacillus reuteri* were reported to have antiproliferative and antimetastatic potential in human stem-like colon cancer cells (HT29-ShE).^[110]^

Another potential therapeutic strategy for CRC is the administration of probiotics in combination with prebiotics to induce the growth or activity of these beneficial microorganisms. For example, *Lactobacillus* and *Bifidobacteria* combined with prebiotics such as oligofructose and inulin can suppress tumor progression. In addition, synbiotics in rats containing *B infantum*, *L acidophilus*, *B bifidum*, maltodextrin (LBB), and oligofructose elevated the expression of intestinal ZO-1, MUC2, TLR-2, and occludin and downregulated COX-2 and TLR-4.^[99]^

*L rhamnosus* GG (LGG) was shown to suppress the expression of β-catenin and Bcl-2 in rats, while upregulating the expression of P53 and Bax, resulting in decreased cell proliferation and carcinogenesis. Decreasing the levels of pro-inflammatory molecules, including COX-2 and NF-κB-p65, is another effect of *L rhamnosus*.^[111]^ Co-administration of *Bifidobacterium* spp. and *Lactobacillus* spp. increases anti-inflammatory cytokines and decreases the levels of genes related to regulatory T cell and Th2 responses,^[112,113]^ which may play a protective role against the initiation phase of cancer. Thus, it can be concluded that the administration of probiotics may help prevent the early stages of CRC and increase the efficacy of other therapeutic approaches such as chemotherapy.^[114]^

In 2012, a probiotic mix composed of 7 different strains of *Lactobacilli*, *Bifidobacteria*, and *Streptococcus* was administered to an azoxymethane-induced CRC mouse model. The results showed a significant decrease in the incidence of carcinogenesis. This phenomenon is due to the modulation of mucosal CD4^+^ T polarization and changes in gene expression.^[115]^ Another study examined the administration of *B infantis* in a CRC rat model and reported a significant decrease in chemotherapy-induced intestinal mucositis due to the suppression of pro-inflammatory cytokines such as IL-6, IL-1β, and TNF-α, as well as increased CD4^+^ CD25^+^ Foxp3^+^ T regulatory cell response.^[116]^ According to data from in vivo studies performed on mice, *Bacteroides* and *Bifidobacterium* contribute to anti-PD-L1 and anti-CTLA-4^[116,117]^ induction of dendritic cells and antitumor T cell response. In 2015, Sivan et al^[117]^ compared the administration of *Bifidobacterium* alone, as well as the anti–PD-L1 therapy. This study showed that the combination of these 2 strategies resulted in maximum efficiency. Co-administration of *B longum* and *B breve* is a novel method for preventing tumor development.^[117]^ The data revealed that, in a BALB/c mouse model, administration of *L lactis* might reduce the amount of H_2_O_2_ and catalase activity, which leads to a decrease in tissue inflammation and colon damage.^[118]^

## 5. The beneficial effects of probiotics through modulating the inflammatory signaling pathways

Intestinal diseases, especially infectious diseases, are recognized as a major health problem in developing countries. However, chronic intestinal diseases are more widespread in developed countries, and their incidence has continued to increase in recent decades in various regions worldwide.^[119]^ The exact pathogenic mechanisms underlying the development of selected chronic bowel diseases remain unclear. The major clinical manifestations of chronic bowel disease are IBDs, necrotizing enterocolitis, and malabsorption syndromes.^[120]^ IBD describes 4 pathologies: ulcerative colitis, Crohn disease, pouchitis, and microscopic colitis. These diseases are systemic disorders that affect the GI tract and often have extraintestinal manifestations in which epithelial barrier function is a critical factor for onset. In addition, innate immunity and commensal intestinal bacteria play important roles.^[121]^

Studies have shown that immune modulation by probiotic bacteria may be due to the release of anti-inflammatory cytokines into the gut. Nevertheless, the specific molecular interactions between probiotics and their hosts are not well defined. *Lactobacillus* and *Bifidobacterium* are the most commonly used probiotics for humans belong to *Lactobacillus* and *Bifidobacterium*. Certain *Lactobacillus* strains can modulate cytokine production by immune cells, and *Bifidobacterium* can induce the acquisition of tolerance. The different regulatory activities of each probiotic strain are related to their structure, spectrum of mediators released, and different signaling pathways that are activated simultaneously.^[18,122]^

A study conducted by Aghamohammad et al showed anti-inflammatory effects on HT-29 cells by modulating the Janus kinase/STAT and NF-kB signaling pathways. They suggested that probiotics of *Lactobacillus* spp. and *Bifidobacterium* spp. as dietary supplements may reduce inflammation-related diseases, such as IBD.^[122]^ In addition, Pagnini et al demonstrated that multiple probiotic formulations prevented intestinal inflammation by locally stimulating the innate immune responses of the epithelium (i.e., increased production of TNF-α from the epithelium and restoration of epithelial barrier function in vivo). They also showed that probiotic bacteria stimulate epithelial production of TNF-α and activate nuclear factor kappa light chain enhancer of activated B cells in vitro. Their results support the hypothesis that probiotics promote gut health by stimulating rather than suppressing the innate immune system.^[123]^

## 6. Conclusion and future prospects

In recent years, interest in the use of probiotic supplements as mediators of health and disease has increased rapidly. This popularity is mainly due to the growing evidence of the interaction between the microbiota and pathophysiological disease processes in humans. In this review, the health benefits, antimicrobial activities, and potential applications of probiotics are discussed. Future research should consider how industry and academia can adapt probiotic research to maximize success. This includes more targeted use of probiotic strains based on individual capabilities as well as the application of several advanced analytical technologies to further understand and accelerate microbiome research. Beneficial microbes can help solve global problems by reducing the risk and impact of disease and removing drugs and toxins from food and the environment. These are exciting times with many career opportunities for probiotic and prebiotic research in the sciences and in applications to many other disciplines.

## Acknowledgments

The authors of this article thank the personnel at the Pasteur Institute of Iran in Tehran, Iran, for their support.

## Author contributions

**Conceptualization:** Mahdi Rohani.

**Data curation:** Amin Sepehr, Seyedeh Tina Miri, Nazanin Rahimirad.

**Writing – original draft:** Amin Sepehr, Shadi Aghamohammad, Mahnaz Milani.

**Writing – review & editing:** Mohammad-Reza Pourshafie, Mahdi Rohani.
